# Prospective clinical study of capecitabine plus oxaliplatin concurrent chemoradiotherapy after radical resection of rectal cancer

**DOI:** 10.1186/s12935-018-0608-x

**Published:** 2018-08-29

**Authors:** Wanghua Chen, Wenling Wang, Yuxin Li, Hongmin Dong, Gang Wang, Xiaokai Li

**Affiliations:** Department of Oncology, Affiliated Hospital of Guizhou Medical University, Guizhou Cancer Hospital, No 28, Guiyi Street, Guiyang, 550001 Guizhou China

**Keywords:** Capecitabine, Chemoradiotherapy, Oxaliplatin, Radical operation, Rectal cancer

## Abstract

**Background:**

To investigate the efficacy and safety of concurrent chemoradiotherapy with capecitabine or oxaliplatin in locally advanced (T3-4/N + M0) rectal cancer.

**Methods:**

56 patients with rectal cancer after radical operation were randomly divided into CAPE-OX-CRT group: capecitabine + oxaliplatin concurrent chemoradiotherapy (30 cases), CAPE-CRT group: capecitabine concurrent chemoradiotherapy (control, 26 cases).

**Results:**

The incidence of grade 1–2 acute toxicity in CAPE-OX-CRT group during concurrent CRT was significantly higher than that in control group, the difference was statistically significant (P < 0.05). Grade 3 toxicities were not statistically significant between the two groups (P > 0.05). No grade 4 toxicity was found in both groups. The incidences of interrupted or suspend concurrent chemotherapy in both groups were 19.23% and 46.67%, respectively, P < 0.05. The incidences of interruption or suspension of radiotherapy were 11.54% and 30% respectively (P > 0.05). The completion rate of adjuvant chemotherapy in control group was higher than that in CAPE-OX-CRT group, but the difference was not statistically significant (P > 0.05). In postoperative adjuvant chemotherapy, the incidence of bone marrow suppression in CAPE-OX-CRT group was higher than that in control group (P < 0.05), and the incidence of non-hematologic adverse reactions was similar between the two groups.

**Conclusion:**

Capecitabine combined oxaliplatin concurrent CRT, and oxaliplatin concurrent CRT have a good effect for treatment of patients with locally advanced rectal cancer after radical resection of rectal cancer.

## Background

Colorectal cancer (CRC) is the third most common malignancy and the fourth leading cause of cancer death in the world, there were approximately 1.4 million new cases and nearly 0.7 million deaths in 2012. At present, in many medium-to high Human Development Index (HDI) countries, especially in Eastern Europe, Asia and South America, the incidence of CRC incidence and mortality are increasing significantly [1, 2]. The number of newly added colorectal cancers in China is as high as 0.13–0.16 million cases each year. The prevalence of colorectal cancer in our country has reached as high as 46.8/100,000. Clinically, local advanced low rectal cancer is more common, accounting for 70–80% of patients with rectal cancer. NCCN clinical practice guidelines in Oncology (Rectal Cancer) [3] preferred recommendation is preoperative concurrent chemoradiotherapy (CRT) for locally advanced colorectal cancer (type I evidence), but concurrent adjuvant radiotherapy for postoperative locally advanced colorectal cancer without concurrent preoperative CRT remains the standard therapy. In recent years, with capecitabine, oxaliplatin in the treatment of advanced colorectal cancer made a breakthrough. Capecitabine and oxaliplatin are both active anticancer agents in the treatment of patients with advanced colorectal cancer. In a previous phase I/II trial, Bajeta et al. [4] demonstrated that capecitabine can safely replace 5-FU in combination with oxaliplatin and irinotecan with promising results in terms of activity. Up to now, there are five randomized clinical trial for preoperative capecitabine + oxaliplatin neoadjuvant concurrent CRT in rectal cancer [5–10]. However, researches on adjuvant chemotherapy combined with radiotherapy after radical resection of rectal cancer have rarely been reported. For esophageal cancer patients treated by oxaliplatin 120 mg/m^2^ intravenously on day 1, and capecitabine 1000 mg/m^2^ orally twice daily on days 1–14 in a 21-day cycle of therapy, nausea and vomiting occurred in 51.6% of patients, leukopenia and diarrhea in 50%, stomatitis in 39.1%, polyneuropathy and hand-foot syndrome occurred in 37.5% of patients [11]. The purpose of this study was to investigate the safety and efficacy of combined chemotherapy of capecitabine-oxaliplatin and oxaliplatin alone when concurrent radiotherapy was performed in patients after radical resection of locally advanced rectal cancer.

## Materials and methods

### Research object

From January 2014 to June 2017, a total of 56 patients undergoing radical resection of rectal cancer were involved in capecitabine combined with oxaliplatin concurrent CRT study.

Inclusion criteria: (1) age ≥ 18 years and KPS ≥ 70; (2) pathological diagnosis of the lower edge of the tumor within 12 cm from the anal verge in the lower segment of II/III rectal adenocarcinoma; (3) no distant metastasis; (4) blood, liver and kidney function, electrolyte and cardiopulmonary function tests were normal; (5) no radiotherapy and chemotherapy contraindications; (6) no history of allergy to fluorouracil drugs and platinum-based drugs; (7) the first treatment; (8) no history of pelvic radiotherapy. Exclusion criteria: (1) patients who did not meet the above criteria were excluded; (2) infection accompanied by any nucleoside analog therapy, known dihydropyrimidine dehydrogenase deficiency; (3) mental disorders or conditions that interfere with oral drug intake compliance. Pregnant or lactating women were also excluded. All patients entered the informed consent form. The study was approved by the Ethics Committee of Guizhou Medical University.

Fifty-six patients were randomly divided into CAPE-OX-CRT group: study group (n = 30, capecitabine + oxaliplatin concurrent CRT group); CAPE-CRT group: control group (n = 26, capecitabine concurrent CRT group). The general clinical data of two groups are shown in Table 1.

**Table 1:** The general clinical data of patients

Information	CAPE-OX-CRT group, n = 30 (%)	Control group n = 26 (%)	*P*
Age (years)			
Median age	58	58	
Range	27–72	24–73	
Gender			0.432
Male	13 (43.33)	14 (53.8)	
Female	17 (56.67)	12 (46.2)	
KPS score			0.240
70	2 (6)	1 (3.85)	
80	22 (74)	20 (76.92)	
90	6 (20)	5 (19.23)	
Postoperative staging			0.890
II	11 (36.67)	10 (38.46)	
III	19 (63.33)	16 (61.54)	
Postoperative T staging			0.096
T2	4 (13.33)	4 (15.38)	
T3	11 (36.67)	9 (34.62)	
T4a	12 (40)	10 (38.46)	
T4b	3 (10)	3 (11.54)	
Postoperative N staging			0.437
N0	5 (16.67)	6 (23.08)	
N1	16 (53.33)	12 (46.15)	
N2	9 (30)	8 (30.77)	

### Radical surgery

All patients underwent Miles surgery [12] and Dixon surgery [13], according to the principle of total mesorectal excision. Postoperative pathology confirmed that all patients achieved R0 resection.

### Postoperative concurrent chemoradiotherapy

Postoperative pelvic radiotherapy combined capecitabine with or without oxaliplatin concurrent CRT. Radiotherapy clinical target area (CTV), including the anastomoic area, the nub rectum, the part of the sigmoid colon, the anterior region of the sacral, the lateral wall of the pelvis, and ischiorectal fossa. The regional lymphatic drainage area included the rectum mesentery area, internal-iliac lymph nodes, or part of the common iliac artery or external blood vessels in and around the lymph nodes and obturator region. CTV upper boundary was under L5 margin, the lower boundaries were under the obturator margin, lateral boundary was the inner edge of the true pelvis, the front boundary was at 1/4–1/3 of the back wall, and the back boundary included half of the sacrum (S3 superior border above) and sacral cortices posterior (S3 superior border below). CTV included the lymphatic drainage area of internal and external sacrum above the level of S3, The planning target volume (PTV) comprised the expanded CTV with 0.5 cm to the left and the right, respectively, and 1.0 cm in the front and the back as well as the head and the foot, respectively. The delineated organs were bladder, small intestine and bilateral femoral heads.

Three dimensional intensity modulated radiotherapy (IMRT), 6 MV X-rays, conventional segmentation were used to create PTV. The prescription dose for PTV was 45–50.4 Gy and 1.8 Gy per time, 5 times per week, a total of 25–28 times [14].

The minimum dose in the PTV is ≥ 93% of the prescribed dose; the highest dose in the PTV is < 115% of the prescribed dose, ≤ 5% of the PTV volume receives ≥ 110% of the prescribed dose. High dose cannot be distributed in the small intestine/anal canal/anastomosis. Limit dose for organ damage: for small intestine/colon, > 35 Gy/≤ 180 cc, > 40 Gy/≤ 180 cc, > 45 Gy/≤ 65 cc and D_max_ < 50 Gy. For the bladder, the limited dose V50 was ≤ 50%; and bilateral femoral heads V50 was ≤ 5%. Where V_x_ represented the proportion of the volume irradiated by X-Gy accounting for the entire volume (%).

CAPE-OX-CRT group: capecitabine 1300 mg/m^2^/daily, orally twice a day, starting from the 1st day of radiotherapy, continuous taking 14 days after stopping 7 days, and then continue to take 14 days; oxaliplatin: The IV dose was 60 mg/m^2^ at weeks 1, 2, 4 and 5 during radiotherapy. Control group: capecitabine: 1600 mg/m^2^/daily, divided into two orally, then using the same CRT procedure as above group [15].

### Postoperative adjuvant chemotherapy

After 2 weeks of concurrent CRT, the mFOLFOX6 regimen adjuvant chemotherapy for 8 cycles was performed:

Oxaliplatin 85 mg/m^2^ IV over 2 h on day 1, 5-FU 400 mg/m^2^ IV on day 1, 5-Fu:2400 mg/m^2^ 24-h continuous IV infusion on days 1 and 2; then CF 400 mg/m^2^ IV on day 1; chemotherapy repeated every 14 days and twice as one cycle.

### Adverse reaction evaluation

Adverse reactions during CRT were evaluated according to the National Cancer Institute Common Toxicity Criteria for Adverse Events, Version 3.0 [16]. Postoperative adjuvant chemotherapeutic toxicity was evaluated according to the World Health Organization’s guidelines [17].

### Follow-up

Two groups of patients completed postoperative concurrent CRT and adjuvant chemotherapy were followed up every 3 months; follow-up items included inpatient and outpatient review and telephone follow-up for medical history, physical examination, chest and abdomen CT, pelvic CT or MR, colonoscopy, tumor markers, and blood biochemistry.

## Statistical analysis

All data are used SPSS17.0 statistical software package for data analysis. The categorical variables between 2 groups were compared using Chi square test; survival was analyzed by Kaplan–Meier method. Log-rank test was used to compare the survival rates of the two groups. P < 0.05 for the difference was statistically significant.

## Results

### Efficacy

**Fig. 1:**
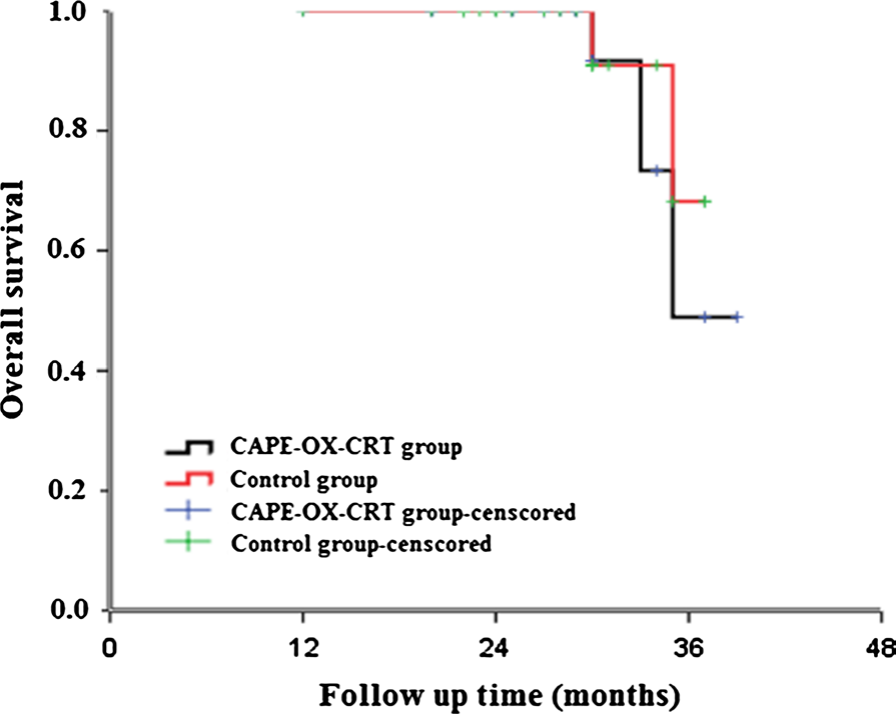
Survival curves of the patients with postoperative adjuvant chemotherapy

The median follow-up time was 30 months (range 3–42 months) with 100% follow-up rate. No local recurrence was observed in both groups within 3 years. The distant metastasis rate of CAPE-OX-CRT group at 3 years was 10% (3/30), while that of control group was 7.70% (2/26), with no significant difference (P = 0.762). There was no significant difference in 3-year overall survival between the two groups (P > 0.05, Fig. 1).

### Adverse reactions of concurrent chemoradiation

In this trial, the main observation was the completion of treatment and the incidence of toxic and side effects in both groups. All patients completed concurrent CRT, while 30% of patients in CAPE-OX-CRT group had toxic side effects during this period, and their chemotherapy dose was reduced. In control group, 7.70% of patients reduced chemotherapy dose due to toxic and side effects during concurrent CRT.

Adverse reactions during CRT in both groups mainly included hematologic toxicity and non-hematologic toxicities. Mostly were grade 1–2 adverse reactions above, grade 3 were rare. Among them, the hematological toxicities mainly manifested in the decrease of leukocytes, neutrophils and platelets. The non-hematologic toxicities mainly include radiation enteritis, digestive tract reaction (nausea and vomiting), and elevated aminotransferases and fatigue (Table 2).

**Table 2:** Toxicities and side effects of concurrent radiotherapy for locally advanced rectal cancer

Toxic reaction	Grade 1–2		*P*	Grade 3		*P*
	CAPE-OX-CRT group (%)	Control group (%)		CAPE-OX-CRT group (%)	Control group (%)	
Diarrhea	14/30 (46.67%)	5/26 (19.23%)	0.028	5/30 (16.67%)	5/26 (19.23%)	0.431
Radiation dermatitis	15/30 (50%)	12/26 (46.15%)	0.774	3/30 (10%)	12/26 (46.15%)	1.000
Bone-marrow suppression	23/30 (76.67%)	10/26 (38.46%)	0.003	3/30 (10%)	10/26 (38.46%)	0.693
Nausea or vomiting	18/30 (60%)	7/26 (26.92%)	0.012	1/30 (3.85%)	7/26 (26.92%)	1.000
Liver dysfunction	9/30 (30%)	2/26 (7.70%)	0.047	0	2/26 (7.70%)	–
Peripheral neurotoxic reactions	6/30 (20%)	1/26 (3.85%)	0.108	0	1/26 (3.85%)	–
Hand**-**foot syndrome	1/30 (3.85%)	0	1.000	1/30 (3.85%)	0	1.000
Fatigue	14/30 (46.67%)	5/26 (19.23%)	0.028	0	5/26 (19.23%)	–

During concurrent CRT, the incidence of interruption or discontinuation of concurrent chemotherapy in CAPE-OX-CRT group was 46.67% due to adverse reactions, in control group was 19.23%, P = 0.028, the difference between the two groups was statistically significant. The incidence of interruption or suspension of radiotherapy due to adverse reactions in both groups was 30% (CAPE-OX-CRT group) and 11.54% (control group), respectively, P = 0.114. There was no significant difference between the two groups.

### Postoperative adjuvant chemotherapy regimen and adverse reactions

Postoperative adjuvant chemotherapy was performed in both groups after concurrent CRT. The median cycle of completion in CAPE-OX-CRT group for adjuvant chemotherapy is 6 cycles (2.0–9.0), in control group was 7 cycles (4.0–8.0). The incidence of grade 1–2 bone marrow suppression in CAPE-OX-CRT group was higher than that in control group, the difference was statistically significant (P < 0.05). The incidence of other remaining 1–4 grade adverse reactions in CAPE-OX-CRT group was higher than that of control group, but there was no significant difference between the two groups (P > 0.05, Tables 2, 3). The completion rate of postoperative adjuvant chemotherapy in CAPE-OX-CRT group was (16/30) 53.33% for 8 cycles, and that in control group was (20/26) 76.92% for 8 cycles. The difference was not statistically significant (P = 0.063).

**Table 3:** Adverse reactions to postoperative adjuvant chemotherapy in both groups of patients

Toxic reaction	Grade 1–2		*P*	Grade 3–4		*P*
	CAPE-OX-CRT group (%)	Control group (%)		CAPE-OX-CRT group (%)	Control group (%)	
Diarrhea	10/30 (33.33%)	7/26 (26.92%)	0.602	5/30 (16.67%)	3/26 (11.54%)	0.712
Bone-marrow suppression	22/30 (73.33%)	10/26 (38.46%)	0.008	4/30 (13.33%)	2/26 (7.69%)	0.675
Nausea/vomiting	17/30 (56.67%)	10/26 (38.46%)	0.173	7/30 (23.33%)	3/26 (11.54%)	0.310
Liver dysfunction	4//30 (13.33%)	2/26 (7.69%)	0.303	0	0	–
Fatigue	20/30 (66.67%)	12/26 (46.15%)	0.121	6/30 (20%)	2/22 (7.69%)	0.263
Peripheral neurotoxic reactions	8/30 (26.67%)	4/26 (15.38%)	0.528	0	0	–

## Discussion

Rectal cancer has become one of the most common malignant tumors in our country. Its incidence is spirally increasing at a rate of 4.2%, far exceeding the international standard of 2%. They are characterized by late stage, high proportion of middle and low rectal cancers and simple surgical treatment is not satisfactory. Some patients with locally advanced rectal cancer underwent radical surgery without neoadjuvant therapy, for this part of patients, postoperative concurrent CRT and postoperative adjuvant chemotherapy is particularly important. The NCCN guidelines recommend [3] capecitabine or 5-fluorouracil chemoradiation as the standard protocol for postoperative treatment of type II/III rectal cancer (type I evidence). Compared with historical postoperative chemotherapy and radiotherapy, standard treatment of total mesorectal excision (TME) and neoadjuvant chemoradiation significantly improved the treatment of local rectal adenocarcinoma. Use of oxaliplatin with fluoropyrimidine therapy has provided survival benefit [18, 19]. Capecitabine is currently the only widely accepted oral 5-fluorouracil (5-FU) prodrug. It is mainly converted to 5-FU in human cancer cells and reduces toxicity. Compared with 5-FU infusion chemotherapy, the incidence of grade 3/4 myelotoxicity is significantly reduced [20]. Oxaliplatin mainly causes DNA damage, prevents the synthesis of DNA and RNA to exert cytotoxic effects, and leads to apoptosis of cancer cells [21]. In order to explore the efficacy and safety of concurrent CRT with capecitabine combined with oxaliplatin in patients with locally advanced rectal cancer, from January 2014 we conducted a phase III clinical trial of concurrent CRT with capecitabine + oxaliplatin or capecitabine after radical surgery for locally advanced rectal cancer, in order to provide safe and effective new program of concurrent CRT for patients with locally advanced rectal cancer after radical operation. This clinical study is one of few randomized studies investigating the use of oxaliplatin in capecitabine-based postoperative concurrent CRT in patients with locally advanced rectal cancer.,

Our research shows that 3-year disease-free survival (DFS), 3-year overall survival (OS), 3-year local recurrence, and 3-year distant metastasis were no significant difference between CAPE-OX-CRT group and control group (P > 0.05).

The addition of oxaliplatin increased the toxicity (the incidences of patients who were interrupted or stopped concurrent chemotherapy or radiotherapy were higher in CAPE-OX-CRT group than in control group). There was significant difference between the two groups in the rate of concurrent chemotherapy suspension (P < 0.05).

Hofheinz et al. [22] studied the effectiveness and safety of the capecitabine instead of fluorouracil in the treatment of locally advanced rectal cancer. Their study showed that capecitabine, compared to 5-fluorouracil groups, has no inferiority in 5-year OS; capecitabine can replace fluorouracil for preoperative or postoperative concurrent CRT in the treatment of locally advanced rectal cancer. The study also showed that capecitabine group had a lower distant metastasis rate (19% vs 28%, P = 0.04). In addition, the CAO/ARO/AIO-04 study [6] showed that the addition of oxaliplatin to fluorouracil-based concurrent CRT improved 3-year DFS in patients with locally advanced rectal cancer (71.2% vs 75.9%; P = 0.03). Our current findings and the study of preoperative concurrent chemoradiation published above indicated that weekly oxaliplatin did not increase 3-year DFS in capecitabine-based concurrent CRT. The reasons may be that the capecitabine is superior to fluorouracil in efficacy and it covers the benefit of oxaliplatin in DFS. However, the median follow-up time in both groups in this study was < 3 years, so the OS and DFS results in this study need further follow-up.

The ADORE and CAO/ARO/AIO-04 studies [5, 6] show that patients with rectal cancer can benefit from oxaliplatin-based adjuvant chemotherapy. In this study, 53.33% of patients in CAPE-OX-CRT group and 76.92% of patients in control group completed adjuvant chemotherapy (P = 0.063). More patients in control group received mFOLFOX6 regimen adjuvant chemotherapy. This may also hide the effectiveness of oxaliplatin in concurrent CRT. A number of preoperative radiotherapy and chemotherapy randomized study [5–7] showed capecitabine monotherapy concurrent CRT in patients with grade 3–4 incidence of toxic reactions was 6.6–15.1%, while in the double-drug group (capecitabine + oxaliplatin), grade 3–4 toxicities were 15.4–36.7%. In our study, the incidence of grade 3 toxicities in group A was 13.33–23.33%, B was 7.69–11.54%, and there was no grade 4 toxicity in both groups. Regarding to the completion of postoperative adjuvant chemotherapy, the completion rate of adjuvant chemotherapy in CAPE-OX-CRT group was lower than that in control group (76.92% vs 53.33%, P > 0.05), but the difference was not statistically significant.

The completion rates of 8 cycles of adjuvant chemotherapy in both groups did not reach 80%, only 50% in CAPE-OX-CRT group, and it may be related to the addition of oxaliplatin during concurrent CRT, resulting in increased postoperative adjuvant chemotherapy toxicity. As more patients refused or interrupted systemic chemotherapy, it is difficult to complete adequate and full course of adjuvant chemotherapy. Whether will this increase the risk of distant metastasis of patients and affect long-term survival needs to be further observed. Future studies should expand the number of cases and longer follow-up.

## Conclusion

Although these two methods are effective in the treatment of locally advanced rectal cancer after surgery, capecitabine combined with oxaliplatin chemotherapy regimen failed to further improve its efficacy. The capecitabine combined oxaliplatin therapy (CAPE-OC-CRT) did not increase the 3-year overall survival (OS) for patients after radical resection of rectal cancer, but increased hematological toxicity and non-hematological toxicity (grade 1–2). As a standard treatment for patients with locally advanced rectal cancer after radical treatment, it is recommended to use capecitabine combined with concurrent radiotherapy alone.

## Abbreviations

CRT: concurrent chemoradiotherapy
CTV: clinical target area
PTV: planning target volume
IMRT: intensity modulated radiotherapy
DFS: disease-free survival
OS: overall survival
